# Waste-Based Volatile Fatty Acids for Fuel and Chemical Production

**DOI:** 10.1021/acsenergylett.5c04340

**Published:** 2026-02-14

**Authors:** Mohammed Tahmid, Hyuck Joo Choi, Amanda Lorraine C. Cruz, Daniela Ferreira-Garcia, Ehsan Abbasi, Gerardine G. Botte, Marta C. Hatzell

**Affiliations:** † School of Chemical and Biomolecular Engineering, 115724 Georgia Institute of Technology, 311 Ferst Drive NW, Atlanta, Georgia 30332, United States; ‡ Institute for Sustainability & Circular Economy, Chemical and Electrochemical Technology and Innovation Laboratory, 6177 Texas Tech University, 2500 Broadway, Lubbock, Texas 79409, United States; ¶ Department of Chemical Engineering, 1372 Texas Tech University, 2500 Broadway, Lubbock, Texas 79409, United States; § George W. Woodruff School of Mechanical Engineering, 115724 Georgia Institute of Technology, 770 Ferst Drive NW, Atlanta, Georgia 30332, United States

## Abstract

Volatile fatty acids (VFAs) are versatile intermediates for circular chemical and fuel manufacturing. Current VFA production relies heavily on fossil feedstocks and serves a narrow set of commodity markets. Here, we project VFA demand through 2050 and evaluate the potential to recover VFAs from abundant, widely available waste streams, exploring how waste-derived VFAs can support a broader circular carbon economy by midcentury. We project that the global VFA recovery potential from waste could reach ∼581 Mt yr^–1^ by 2050, roughly 10 times higher than projected demand in existing markets. Integrating waste-carbon streams with anaerobic digestion infrastructure and emerging VFA production and separation technologies provides a feasible route for large-scale waste-to-VFA conversion. We project that in a 2050 circular scenario, ∼581 Mt yr^–1^ of waste-derived VFAs can be flexibly distributed across various chemicals and energy sectors, supplying a significant portion of anticipated demand and establishing VFAs as a scalable, multifunctional platform molecule for a circular carbon economy.

## Graphical abstract

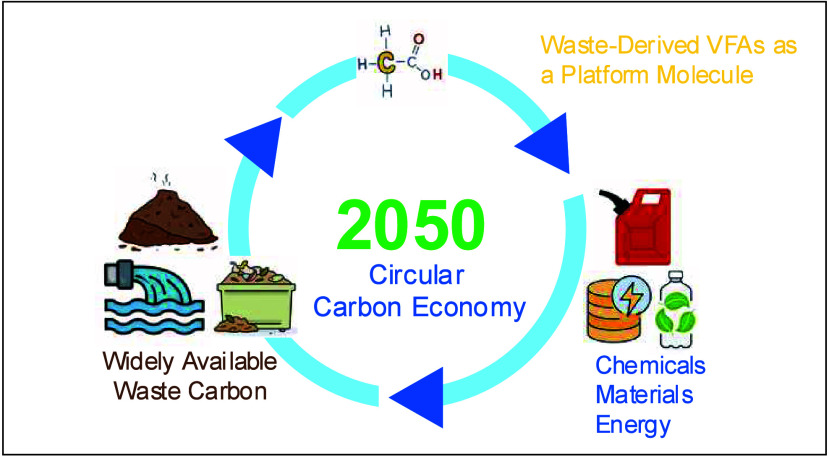

Volatile fatty acids (VFAs) are a class of short-chain carboxylic acids (C_2_–C_6_), with acetic, propionic, and butyric acids most extensively produced industrially.^1^ Once viewed as transient intermediates of anaerobic fermentation, VFAs are now recognized as versatile carbon platform used in chemical, pharmaceutical, food, and agriculture industries.^2^ VFAs also hold promise as feedstocks for synthetic aviation fuels, polymers, and other value-added products.^3–7^ This evolution reflects a broader shift from linear waste treatment toward circular value recovery. In this model, organic residues from municipal, agricultural, and industrial streams are transformed into high-value chemicals and energy, positioning VFAs as key intermediates in the transition to a sustainable, circular carbon economy.^8^

Interest in VFAs is driven by the dual pressures of rising carbon prices^9^ and the growing focus on waste valorization.^10^ The degradation of organic matter during wastewater treatment alone contributes ∼1.6% of global greenhouse gas (GHG) emissions and ∼5% of global noncarbon dioxide emissions.^11,12^ Converting organic waste into VFAs offers a pathway to mitigate these emissions while simultaneously producing economically valuable products, establishing VFAs as circular carbon carriers within the emerging bioeconomy.

Currently, ∼90% of VFAs are produced via petrochemical routes,^13^ which are associated with substantial fossil fuel use and GHG emissions. This underscores the potential for alternative production pathways. Here, we examine opportunities in both existing and emerging VFA production technologies that can support a circular carbon economy. We project global VFA demand through 2050, considering associated energy requirements and GHG emissions, and assess the global recoverable potential of VFAs from diverse waste streams. We further explore how this potential can grow over time due to population expansion, increasing waste generation, and industrial development. We have also constructed a 2050 circular scenario in which waste-derived VFAs can be flexibly allocated across multiple sectors, including commodity chemicals, fuels, polymers, energy storage, and agriculture, collectively satisfying a substantial fraction of projected market demands in each sector. Through these analyses, we demonstrate how VFAs can serve as central carbon intermediates, actively linking sustainable resource recovery with chemical, energy, and materials production.

## Current Manufacturing of Volatile Fatty Acids

The current industrial production of acetic, propionic, and butyric acids relies on petrochemical oxidation, which accounts for ∼90% of total VFA production. Industrial processes for producing acetic acid are primarily dominated by methanol carbonylation and the oxidation of hydrocarbons, such as acetaldehyde, ethylene, *n*-butane, and naphtha.^14,15^ Propionic acid is synthesized from the catalyzed hydroxy carboxylation of ethylene via rhodium and nickel-based catalyst.^15^ Butyraldehyde is produced via the oxo-synthesis of propylene, which in turn is oxidized to produce butyric acid.^16^ These routes are technologically mature and have high yields and selectivity. However, these processes are also energy and carbon-intensive and rely on nonrenewable feedstocks.

Biobased routes for VFA production often rely on anaerobic fermentation, and account for ∼10% of total VFA production.^13,17,18^ There has been growing interest in alternative biological routes for VFA production, especially with the growth and deployment of anaerobic digestion (AD) at wastewater treatment sites. AD is a scalable fermentation process commonly used to break down waste organic matter (e.g., sludge). Traditionally, AD proceeds through four stages: hydrolysis, acidogenesis, acetogenesis, and methanogenesis.^19^ Most AD systems thus are tuned to generate methane, which can be used as energy.^20^ However, VFAs are produced as intermediates during the acidogenesis and acetogenesis. Most VFAs are subsequently converted during methanogenesis to methane and biogas. Methane produced during anaerobic digestion can be utilized in multiple ways, including on-site heat and power generation, upgrading for injection into natural gas grids, or use as a transportation fuel.^21–24^ In many systems, recovered methane is also recycled internally to meet process heating demands.^25^ However, in pathways targeting VFA accumulation and recovery, methanogenesis must be intentionally inhibited to retain carbon in the liquid phase rather than converting it to biogas.^26–28^

VFAs can be biologically produced via two routes: pure-culture and mixed-culture fermentation processes (Figure 1). Pure-culture systems use specific microbial strains to synthesize pure VFAs that offer high selectivity and well-defined metabolic pathways.^29,30^ Industry has commercially produced acetic acid using *Acetobacter*, *Gluconacetobacter*, and *Gluconobacter*.^31^ Fermentation processes produce butyric acid from sugars, with *Clostridium butyricum* as the most prevalent bacterium.^32–36^ The biobased production process for propionic acid production utilizes bacteria from the genus *Propionibacterium*.^37^ However, these processes are expensive and difficult to scale up due to the high cost of raw materials and the need for sterile operating conditions.^29,30^

**Figure 1:**
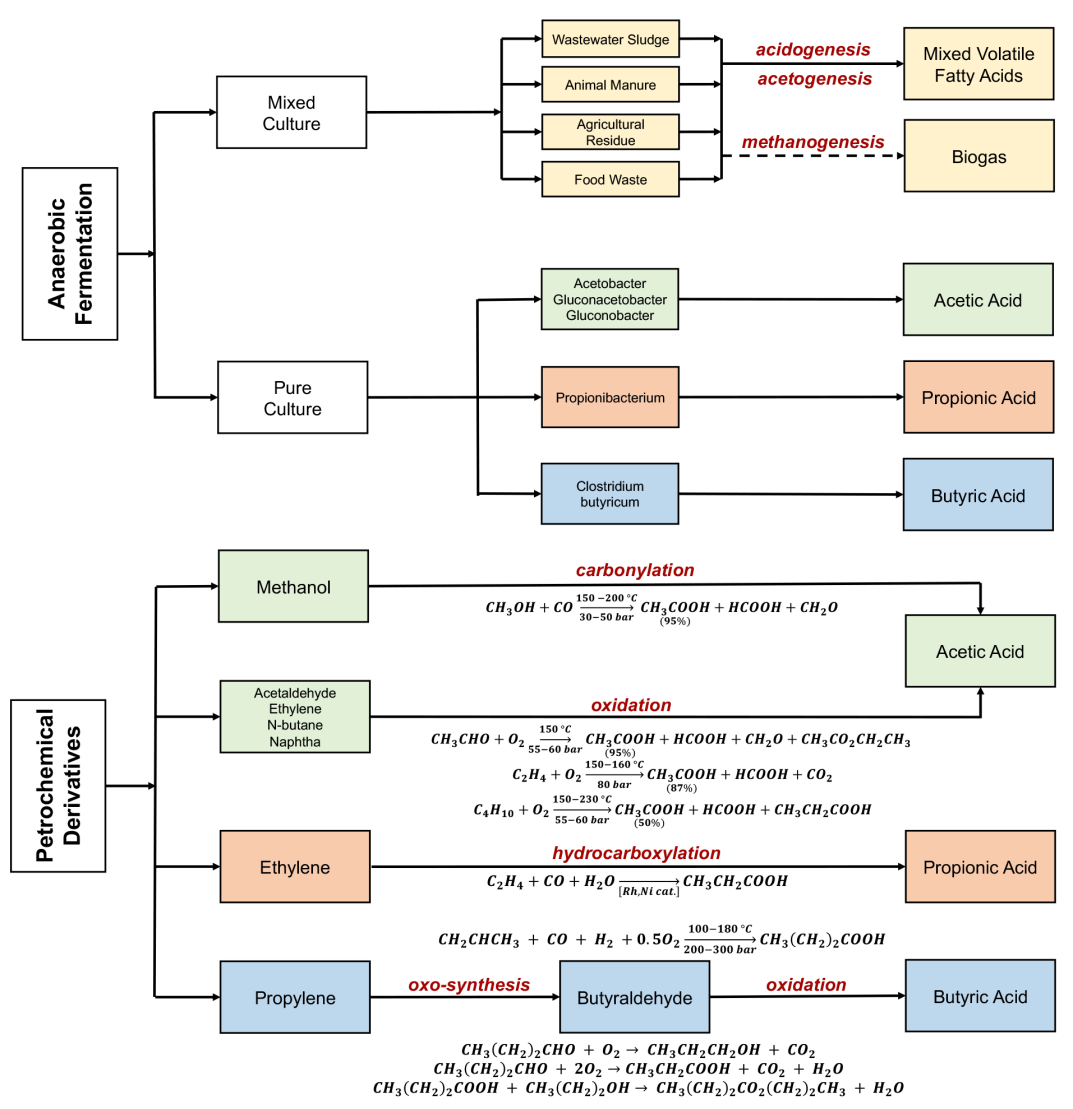
Overview of current processes for VFA manufacturing (based on literature reports).^15,45,53,54^

Mixed-culture fermentation utilizes diverse microbial strains to convert organic-rich wastes such as agricultural residues, food waste, wastewater sludges, and animal manure as alternative carbon sources.^38–41^ The waste streams undergo acidogenesis and acetogenesis to convert complex substrates to VFAs mainly composed of acetic, propionic and butyric acid under nonsterile conditions.^19^ The process enables waste valorization by utilizing renewable and low-cost feedstocks, thus reducing the dependence on pure carbon substrates.^42,43^ However, current routes yield mixed VFA streams with variable compositions increasing the costs associated with downstream recovery.^44,45^ Different pretreatment methods have been studied, such as physical, ultrasonic, thermal, chemical, and thermochemical to maximize the production of VFAs and minimize methane generation.^46–51^

In terms of industrial practices, petrochemical oxidation remains commercially mature, although the environmental footprint of these processes highlights the need for sustainable alternatives.^13^ Biological processes are currently at pilot to demonstration scales, and address the need for lower energy demands and nonreliance on depleting resources by utilizing low-value organic wastes.^13^ Mixed-culture biological processes can accommodate heterogeneous waste feedstocks with varying compositions across sources due to the presence of diverse microbial consortia. However, the production rate of these processes remains limited, as VFA generation is constrained by hydrolysis- and acidification-limited kinetics, product inhibition, and the need for multiday sludge retention times, resulting in relatively low volumetric productivities compared to petrochemical routes.^52^ Consequently, biological routes remain limited as a result of low selectivity and process instability compared with fossil-based synthesis.

## Future Manufacturing of Volatile Fatty Acids

Building on the limitations of current petrochemical and biological production routes, future VFA manufacturing aims to improve sustainability by increasing yields, reducing energy demand, and lowering carbon footprints. Researchers are examining ways to process intensify anaerobic fermentation to suppress competing pathways. In addition, significant efforts to improve process intensification have centered on integrating advanced separation and electrochemical processes (Figure 2).^44^ Hydrolysis limits the rate of acidogenic fermentation because hydrolysis controls the supply of readily accessible substrate carbon.^55^ Hydrolysis is often the bottleneck process for anaerobic fermentation rate and has been verified kinetic models.^56,57^ Hydrolysis kinetics could be more limiting when the substrates are rich in proteins or other difficult to degrade substances.^1^ Operational parameters, including temperature, pH, feedstock composition, and feedstock pretreatment, strongly influence hydrolysis kinetics and require optimization alongside downstream acidogenesis conditions.^44^ Enhancing acidogenesis plays a critical role in maximizing VFA yields. Key factors include substrate characteristics, inoculum selection, feedstock pH, and temperature. Higher total Kjeldahl nitrogen (TKN) concentrations increase propionate production by 78%, whereas substrates with excessively high protein content (C/N > 7.1) limit propionate production.^55^ A lower C/N ratio could correlate to the existence of ammonia or ammonium, effectively suppressing acidogenesis.^1^ Thus, both hydrolysis and acidogenesis are crucial steps for VFA production because hydrolysis governs the rate of VFA production and acidogenesis dictates the selectivity of VFAs.

**Figure 2:**
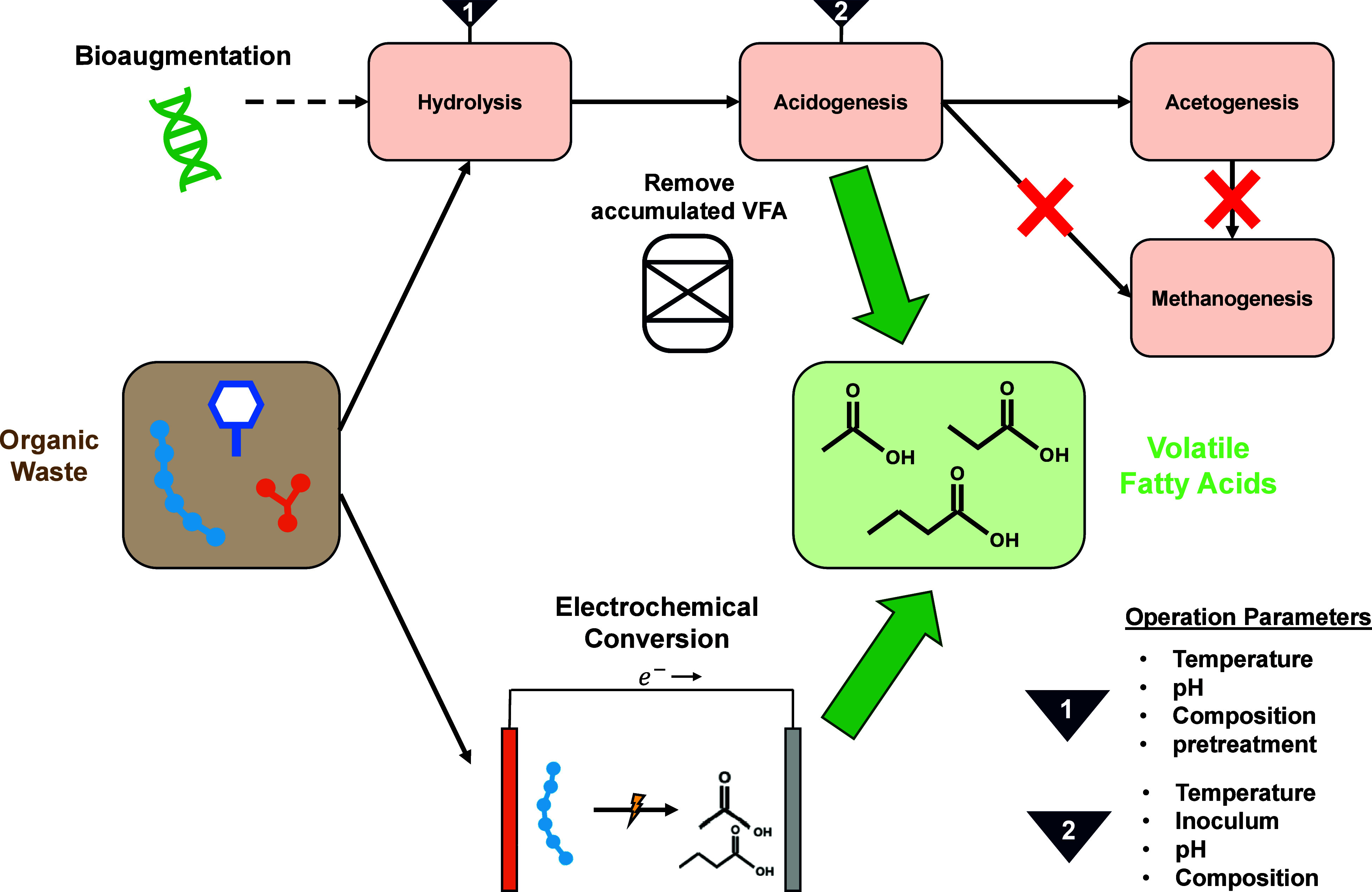
Overview of future biological and electrochemical manufacturing routes for VFA production.

Codigestion of complementary feeds can help optimize VFA production by balancing such substrate composition issues.^58^ Precise pH control can both suppress unwanted methanogen reaction (favored between pH of 7.8 and 8.2) and affect the activity of microorganisms in acidic (pH < 3) or alkaline (pH > 12) environments.^59,60^ Bioaugmentation is a strategy that introduces target microbial strains into a system to selectively enhance hydrolytic and acidogenic kinetics.^61^ For instance, *C. butyricum* increased butyric acid production by 20% in food waste fermentation,^62^ while homoacetogenic bacteria increased acetic acid production by 70%.^63^ Introduction of specific microbial strains also improves substrate hydrolysis by producing enzymes active in cold temperatures, addressing the decreased hydrolysis rate and low temperatures.^64^ However, challenges remain in ensuring the persistence, activity, and dominance of introduced strains and reducing the cost of cultivation at scale. Even with resilient microbial strains, the product VFA profile strongly depends on the feedstock composition microbial competition, requiring careful control per manufacturing site.^1,2^

As VFAs accumulate, acidogenesis becomes thermodynamically unfavorable as the metabolic pathway shifts toward competing products and reduces the total yield of VFAs.^65^ To maintain high VFA conversion and prevent excessive accumulation, product separation is required. Conventional separation methods such as precipitation, extraction, crystallization, and distillation prevent excessive VFA accumulation but often produce unwanted solids, require high chemical usage, and deliver low yields with high energy consumption.^66^ One promising VFA separation route that avoids VFA accumulation is pressure-driven membrane filtration technologies such as reverse osmosis, microfiltration, and nanofiltration. Pressure-driven filtration methods present high separation performances up to 97%, scalability, and low energy demand for VFAs in high concentration and purity.^67,68^ However, rejection performance reduction due to concentration polarization, membrane fouling, and low VFA concentrations in a complex waste mixture are essential bottlenecks to scaling filtration methods for VFA separation. Adsorption through ion exchange resins have also been tested for VFA separation for their cost-effectiveness, scalability, and selectivity.^69^ Ion exchange resin has shown over 98% adsorption for hexanoic acid and reached 99% for synthetic mixture of VFAs.^70^ However, fouling by proteins and solids, necessity of chemicals for regeneration, and resin cost limit adsorption from being widely used. Another alternative approach is electrodialysis (ED).^71,72^ ED offers high separation efficiency, low pollution, and generation of acid and base conditions.^73,74^ Bipolar membrane electrodialysis (BMED) coupled with fermentation recovered 50–70% of carboxylates from *E. coli* fermentation product mixtures^75^ while a standard ED configuration achieved 99% removal from fermentation broths.^73^ However, membrane fouling and sorption of VFAs onto the membrane inhibit separation performance and increase membrane cleaning and exchange costs.^76^

Electrochemical oxidation presents a fundamentally different route for manufacturing VFAs directly from organic wastes such as sludge and biomass (Figure 2). Advanced oxidation processes using reactive oxygen species (ROS) can substitute the hydrolysis step using electrocatalysts such as boron-doped diamond (BDD). BDD produces hydroxyl radicals, which can break down complex structures such as lipids, carbohydrates, cellulose, and proteins to shorter carbon strings.^77^ However, prolonged exposure of long chains to ROS risks overoxidizing intermediates beyond VFAs.^78^ This overoxidation challenge reflects competing reaction pathways: while hydroxyl radicals and other reactive species facilitate substrate breakdown, these same radicals can continue oxidizing VFAs intermediates once formed and on the surface of the electrode. The balance between VFAs formation and its mineralization depends on many factors, among which electrode material selection is one of the most important for further optimization. As different electrocatalysts exhibit different catalytic performances and selectivities, recent advancements illustrate these behavior; copper electrodes produced 450 mg/L VFAs from activated sludge with 30% volatile solid removal,^79^ while Ti/RuO_2_–IrO_2_ dimensionally stable anodes produced 69 mg/L VFA concentration from cashew nutshell waste.^80^ Comprehensive understanding of the electrochemical conversion process from waste to VFAs is still required, specifically in terms of mass transport, thermal effect, and electrolyte-electrode interactions.^80^

In addition to catalytic selectivity, electrode stability must also be considered. BDD exhibits exceptional stability across a wide range of operating conditions, including high current densities, highly alkaline or acidic environments, and elevated temperatures, making it particularly suitable for VFA production.^81^ Other electrodes, such as copper, nickel, and iron-based materials, require further investigation for their long-term stability and resistance to oxidative degradation. For instance, fragmentation of amino acids during oxidation releases amine groups that can form ammonia, a strong complexing agent for many metals.^82^ This complexation can accelerate corrosion or dissolution of the electrode surface, thereby compromising its stability and performance.^83^ Among the many advantages of electrochemical oxidation, the complexity of the feedstock remains one of the main challenges. The composition of the feed stream can vary significantly, which directly affects process performance. In addition, impurities such as dissolved metals and anions (e.g., chloride ions) may influence electrode stability and reaction selectivity, and may also lead to unintended environmental concerns, including metal speciation and the formation of chlorinated byproducts. To address these challenges, we propose several mitigation strategies. These include appropriate pretreatment of the waste stream to reduce compositional variability and remove problematic impurities, as well as the integration of artificial intelligence-based control systems to dynamically adjust operational parameters (e.g., voltage and current density) and guide catalyst design (including catalyst formulation and synthesis procedures). Such approaches can enhance reaction selectivity and support rational electrode and catalyst development.

Hybrid systems such as microbial electrochemical technologies (MET) transform organic matter to VFAs by using electrodes to redirect electron flow away from methanogenesis and toward hydrolytic and acidogenic pathways.^44,84,85^ For example, microbial fuel cells constructed with a photothermal Janus anode equipped with a waterproof nanoporous layer with an electroconductive porous layer prevented algal growth by 1.6-fold while improving electron transfer by 24-fold.^86^ Coupling microbial electrosynthesis with an electrolytic bubble column helped achieve acetate concentrations up to 34.5 g/L.^87^ However, MET faces major obstacles such as biofouling, low efficiency, and inconsistent operation when scaled.^88–90^ Further research is required in electrode and membrane design for stable and efficient operation.

We note that biological and electrochemical VFA production routes differ substantially in technological maturity. Conventional anaerobic fermentation for mixed VFA production is commercially implemented and operates at high technology readiness levels (TRL 8–9), whereas process-intensified biological systems including bioaugmentation^61,62^ and integrated separations^73,75^ generally fall within TRL 4–6. In contrast, electrochemical oxidation and MET^77,79^ remain at early stages of development (TRL 2–4), with key challenges related to selectivity, electrode stability, energy efficiency, and scale-up. Distinguishing these TRL differences is critical when evaluating the feasibility and near-term deployment potential of emerging VFA manufacturing pathways. To address immediate demand for VFA resources, VFA manufacturing must be integrated with existing wastewater treatment infrastructure, specifically anaerobic digesters, to utilize waste VFA resources. Maximizing VFA yields must be connected with selective separation of VFAs to ensure pure manufacturing of VFAs. In the long term, electrochemical oxidation will provide a sustainable route for manufacturing VFAs from organic wastes, eliminating days and weeks of organic waste disintegration. These approaches point toward a new generation of sustainable VFA production routes and enable continuous, tunable production from diverse waste streams.

## Current and Future Market and Energy Demands of Volatile Fatty Acids

VFAs represent a rapidly expanding segment of the global carbon economy. We analyzed current and projected VFA market trends to assess economic and energetic relevance through 2050. The total global VFA market volume was ∼19 Mt yr^–1^ in 2024, dominated by acetic acid (>95%), with smaller contributions from propionic and higher (C4+) acids. Based on this baseline, we project total market volume to nearly triple to ∼60 Mt yr^–1^ by 2050 (Figure 3a), remaining dominated by acetic acid. This continued dominance stems from the central role of acetic acid in producing vinyl acetate monomer for paints and coatings, growth in the pharmaceutical sector, and rising consumer demand for processed foods and vinegar.^91^ We projected future VFA market trajectories using compound annual growth rates (CAGR) from recent industry analyses, reflecting expected growth across chemical, material, pharmaceutical, agricultural, and food applications. Our projection assumes a CAGR of 4.4% for acetic acid,^91^ 2.7% for propionic acid,^92^ and ∼10% for C4+ acids.^93,94^

**Figure 3:**
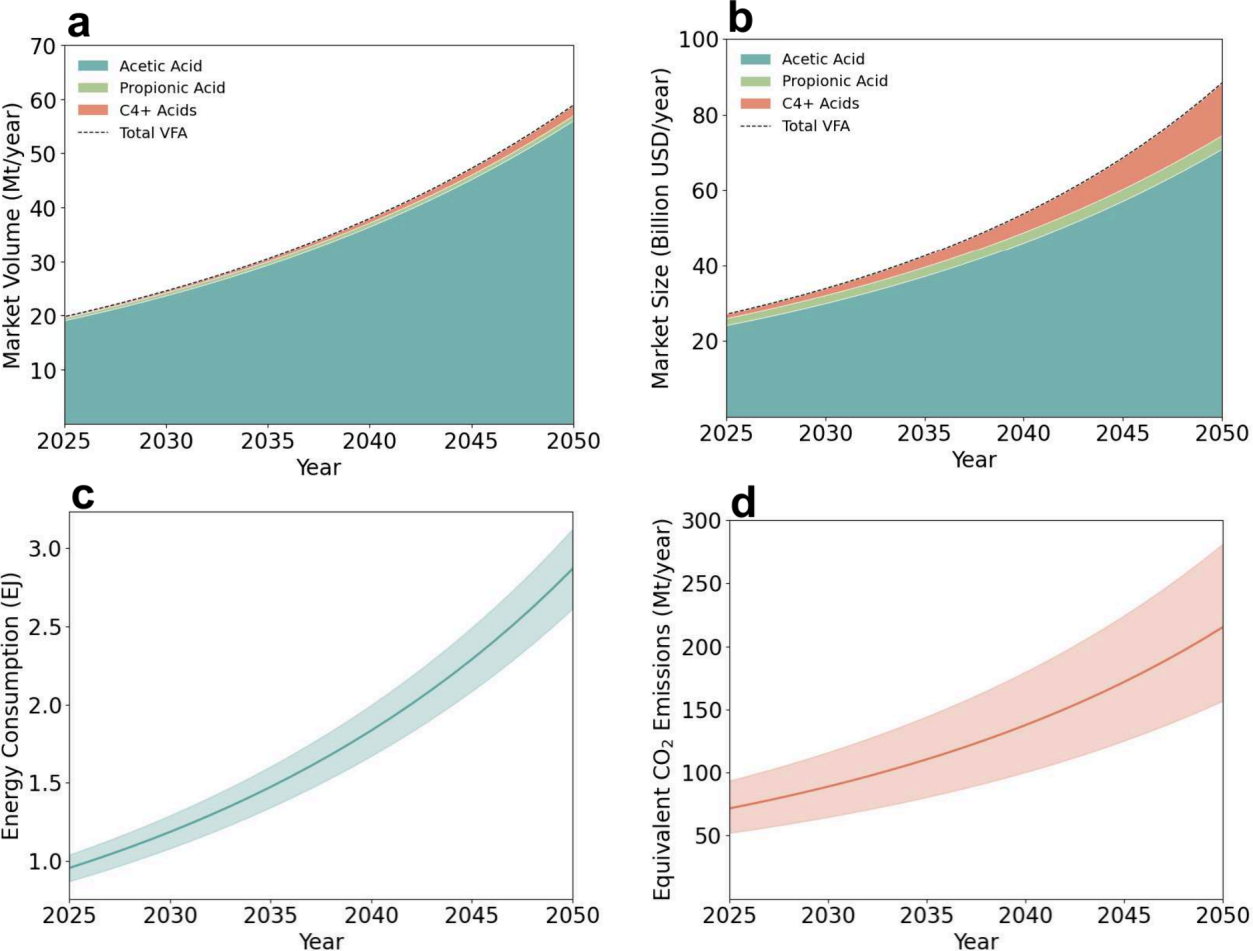
Global trajectories of VFA production and associated impacts from 2025 to 2050. (a) Projected VFA market volume (Mt yr^–1^), (b) corresponding global market value (billion USD yr^–1^), (c) estimated primary energy demand associated with VFA production (EJ), and (d) equivalent CO_2_ emissions. For (c) and (d), the solid lines represent baseline estimates, while the shaded area indicates the uncertainty range, bounded by the low and high scenarios.

Propionic acid growth remains comparatively modest, driven mainly by established use as a preservative and additive across food, feed, and pharmaceutical sectors.^92^ The faster growth of C4+ acids reflects increasing consumption of butyric acid in animal nutrition and pharmaceuticals,^93^ and of valeric acid in agricultural, automotive, and specialty chemical applications.^94^ The corresponding market size follows a similar trajectory, rising from ∼25 billion USD yr^–1^ in 2024 to ∼90 billion USD yr^–1^ by 2050 (Figure 3b), assuming current price differentials and demand elasticity persist. Although acetic acid continues to dominate the total market size, the higher value C4+ segment is expected to account for a progressively larger share of total market value.

The energy intensity of VFA production depends strongly on the carbon source and synthesis route. Conventional petrochemical oxidation of hydrocarbons to VFAs requires 44–53 GJ t^–1^ energy,^95,96^ driven almost entirely by fossil-based feedstocks. Using these benchmarks, the total energy demand for VFA production in 2025 is estimated at ∼0.95 EJ yr^–1^, with corresponding CO_2_ emissions of ∼72 Mt yr^–1^ (∼0.13% of current global GHG emissions) (Figure 3c,d). Under a business-as-usual trajectory and assuming only incremental process improvements, total energy consumption can rise to ∼3 EJ yr^–1^ by 2050, with associated emissions approaching 215 Mt CO_2_ yr^–1^, or about ∼0.4% of projected global emissions. This footprint exceeds that of the world’s most-shipped chemical, methanol (∼165 Mt CO_2_ yr^–1^),^97^ highlighting the emerging energy and climate relevance of VFAs within the global chemical sector.

We highlight that the current petrochemical-based VFA manufacturing processes can consume nearly 3 EJ yr^–1^ and emit over 200 Mt CO_2_ yr^–1^ by 2050, emphasizing the need for sustainable, waste-derived routes supported by advances in bioconversion and separation technologies.

## A Carbon Economy around Volatile Fatty Acids

The vision of a circular carbon economy is rooted in the principles of green chemistry, specifically waste prevention and the use of renewable feedstocks.^98^ VFAs embody these principles by transforming waste organics into reactive carbon intermediates that can be directly reintegrated into chemical and energy value chains. In advancing a circular carbon economy, maximizing the recovery and reuse of all available waste streams is essential. Heterogeneous organic wastes such as municipal solid waste, food waste, and agricultural residues represent a currently under-valorized global potential exceeding one billion tonnes annually.^99^ At present, these streams are primarily treated via composting or AD. While AD is traditionally designed for pathogen reduction and volume minimization, AD also represents a latent biorefinery platform capable of producing VFAs as key intermediates.

Most AD facilities are currently optimized for methane production, however, these systems inherently generate VFAs as intermediates and already operate across a wide range of feedstocks and scales, from small farm units to large urban facilities^100^ making them attractive platforms for alternative valorization strategies. Scalability of AD offers significant potential for regional adaptation, with performance largely shaped by local feedstock availability and collection infrastructure. To assess the magnitude of this opportunity, we estimated the global recoverable potential of VFAs across five representative waste streams, including household wastewater, industrial wastewater, municipal solid waste, agricultural residues, and livestock manure, all of which are characterized by high organic content conducive to VFA generation. Recognizing variability in organic matter hydrolysis kinetics, VFA conversion yields, and separation efficiencies, we considered conservative, baseline, and optimistic scenarios (values and assumptions in Supporting Information, Table S3). Under the baseline scenario, recoverable VFAs from household and industrial wastewater alone total 11.4 Mt·yr^–1^, equivalent to ∼60% of current global VFA demand (Figure 4a). These two streams were prioritized because ∼1,269 wastewater treatment plants in the United States (∼8% of ∼16,000 total)^101^ already operate anaerobic digesters on-site, enabling near-term recovery.

**Figure 4:**
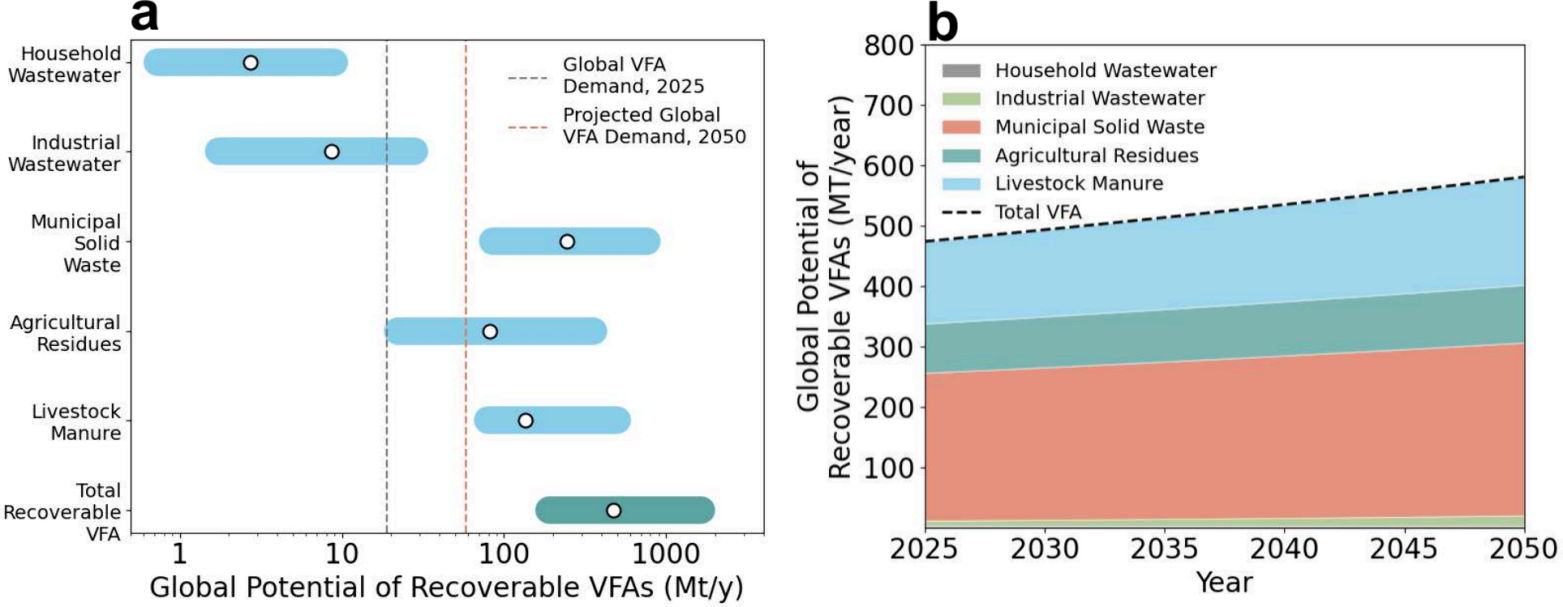
Global potential of waste-derived VFAs. (a) Estimated recoverable VFAs from major waste streams under conservative, baseline, and optimistic recovery scenarios. Ranges reflect variability in hydrolysis efficiency, VFA yield, and separation recovery efficiency across feedstocks, (b) Projected evolution of global recoverable VFAs (2025–2050), accounting for population growth, waste generation, and industrial intensification.

In contrast, municipal solid waste, agricultural residues, and livestock manure dominate the global recoverable VFA resource base, together accounting for nearly 98% of total potential (Figure 4a), due to larger organic content and higher conversion yields under optimized digestion and separation. Aggregating all five feedstocks yields a global recoverable potential of 189 Mt·yr^–1^ (conservative), 475 Mt·yr^–1^ (baseline), and 1,656 Mt·yr^–1^ (optimistic) (Figure 4a). This represents a massive untapped carbon pool, indicating that VFAs are feedstock-abundant but infrastructure-limited. Even accounting for logistical constraints and conversion inefficiencies, the available resource base substantially exceeds foreseeable demand of 60 Mt·yr^–1^ in 2050. We further projected the evolution of recoverable VFAs through 2050, accounting for expected population growth, waste generation trends, and industrial intensification (growth rates and assumptions described in Supporting Information, Table S4). The global VFA recovery potential can approach ∼581 Mt·yr^–1^ in 2050 under baseline assumptions (Figure 4b), which is roughly 10-fold higher than the projected 2050 global VFA demand (∼60 Mt·yr^–1^). This surplus highlights that VFAs have the potential to be a core carbon intermediary in a future circular economy. We emphasize that this estimate represents an upper bound on carbon availability rather than a deployable supply under current infrastructure. Actual recovery will be constrained by competing waste uses, the spatial distribution of feedstocks, logistical limitations, and the pace of separation and upgrading infrastructure deployment.

The global recovery potential of waste-derived VFAs can reach ∼581 Mt yr^–1^ by 2050, enabling flexible use across diverse chemical and energy sectors and supporting a scalable circular carbon economy.

Currently, the global VFA supply chain is dominated by petrochemical production, contributing ∼90% of total output in 2025 (Figure 5). Waste-derived processes, primarily AD of mixed organic wastes, account for the remaining ∼10%, constrained not by resource scarcity but by the lack of systematic valorization of intermediate VFAs within existing AD infrastructure, where methanogenesis remains the terminal metabolic pathway. Arresting methanogenesis and redirecting carbon flux toward VFA accumulation represents a crucial opportunity for near-term carbon circularity. By 2050, however, a fully biogenic VFA economy is technologically feasible, wherein all VFAs could be derived from renewable and waste-based carbon, fully displacing fossil feedstocks.

**Figure 5:**
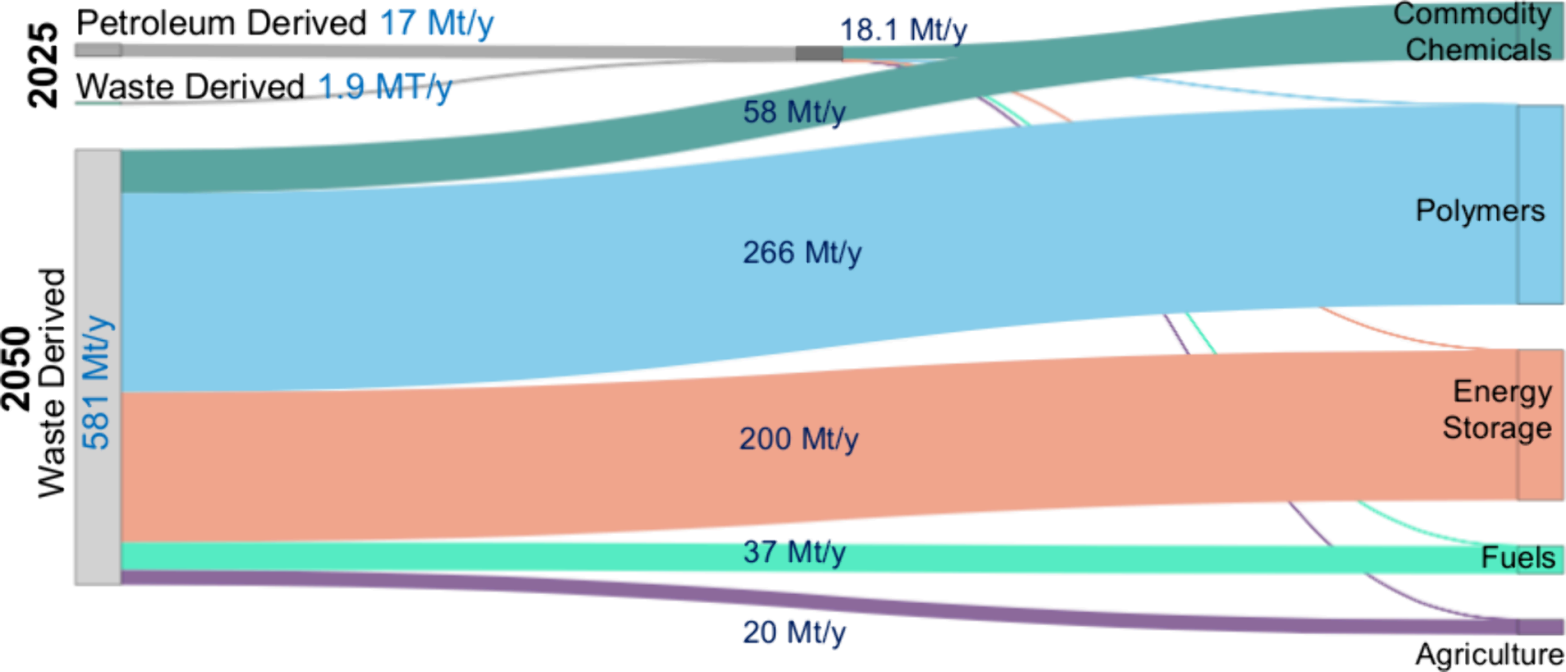
Sankey representation of global VFA flows in 2025 and a projected circular scenario for 2050. In 2025, ∼90% of VFAs originate from petrochemical routes and are primarily used as commodity chemicals (>95% of market). By 2050, all VFAs could be recovered from waste-derived carbon (∼581 Mt yr^–1^), enabling redistribution across diversified markets. Flow widths represent mass flow (Mt yr^–1^).

In 2025, VFAs are used predominantly (∼95%) as commodity chemicals serving as solvents, preservatives, and intermediates in ester and polymer synthesis. The remaining fractions support pilot-scale applications in biofuels, bioplastics, and nutrient recovery. Looking ahead to 2050, a fully waste-derived VFA system could redistribute the entire carbon throughput across multiple high-value applications in a circular carbon economy. Biopolymers like polyhydroxyalkanoates (PHAs) can replace petrochemical plastics due to their biodegradability and strong material properties. VFAs are attractive feedstocks for PHA production because they are direct metabolic precursors of PHAs and can be cheaply sourced from waste, reducing production costs.^3,4^ Catalytic upgrading of VFAs enables production of sustainable aviation fuels (SAFs) with 165% less GHG emissions compared to fossil jet fuel.^5^

In energy storage, VFAs can act as liquid electrochemical intermediates, storing electricity generated from renewable sources in their chemical bonds. This allows renewable energy to be released on demand, either as electricity or as a fuel, effectively serving as a flexible carbon–energy buffer that helps balance supply and demand in renewable energy systems.^6^ VFA-derived soil conditioners and biostimulants enhance nutrient use efficiency and recycle carbon and nitrogen to soils,^7,102^ extending circularity to agriculture. We have constructed a 2050 scenario (Figure 5) based on forecasted sectoral demands, where ∼10% of global recoverable VFAs (∼581 Mt·yr^–1^, baseline scenario) could be directed to commodity chemicals, ∼6% to fuels (particularly SAF), ∼46% to polymers (particularly PHA), ∼35% to energy storage, and ∼3% to agricultural applications. These allocations illustrate how a circular carbon economy could work with waste-derived VFAs collectively satisfying 34–100% of projected market demands in each sector (Supporting Information, Table S5). This shift signifies a redesign of the carbon economy, where waste carbon serves as a flexible, dispatchable feedstock across chemical, energy, and agricultural systems. Importantly, our allocations of VFAs across end-use sectors reflect optionality rather than a prediction of dominant near-term deployment pathways. Polymers, commodity chemicals, and SAFs represent the most credible near and midterm sinks for waste-derived VFAs, given their technological maturity and established markets. In contrast, the use of VFAs as energy storage media remains at an earlier stage of development and is not yet commercial at scale. The inclusion of energy storage in the 2050 scenario is therefore intended to highlight long-term system flexibility and carbon–energy coupling potential, rather than to imply imminent market dominance. Although VFAs exhibit substantially lower energy densities than conventional energy carriers such as hydrogen, methane, or methanol, particularly in dilute waste-derived streams, they are better suited as electrochemical intermediates than as primary energy storage media. VFAs can enable carbon retention, temporal energy shifting, and integration with downstream upgrading, functioning as buffered carbon–energy vectors in circular systems.

Realizing this vision, however, depends on overcoming a number of bottlenecks in VFA production and recovery, particularly within biological conversion pathways. Conventional AD faces several limitations, including low hydrolysis rates, modest VFA yields, unstable acid compositions, and complex downstream purification requirements.^103^ In typical digesters, VFAs and ammonium (NH_4_^+^) accumulate as organic matter is degraded,^104,105^ which can inhibit microbial activity and reduce conversion efficiency.^106^ The digestate matrix further complicates recovery due to its mixture of salts and neutral organics.^107^ Cofermentation of multiple organic waste types has been shown to alleviate inhibition, enhance microbial diversity, and balance nutrient availability, collectively improving process stability and VFA productivity.^57^ Coupling AD or cofermentation with selective separation of the produced VFAs can mitigate inhibition and enable continuous product extraction. Emerging electrochemical–biological hybrids, such as sludge electrolysis using transition-metal catalysts (Cu, Ni, stainless steel) at <2.5 V,^79,108^ offer faster and more energetically efficient VFA generation.

Leveraging existing anaerobic digestion infrastructure and emerging electrochemical–biological systems can unlock diverse waste streams, enabling VFA recovery and upgrading through distributed regional hubs in a circular carbon economy.

Another critical challenge for translating recoverable VFA potential into realized supply is logistics. VFAs produced in anaerobic digesters are typically dilute and geographically distributed, making long-distance transport to centralized manufacturing facilities energetically and economically unfavorable. This constraint motivates decentralized or regional upgrading strategies, in which VFAs are either concentrated, converted to higher-value products, or utilized on-site or near the point of generation. Such distributed manufacturing paradigms shall reduce transportation costs, mitigate handling challenges associated with dilute organic acids, and align naturally with the existing spatial distribution of waste treatment infrastructure.

Direct comparison of production costs across conventional petroleum and waste-derived VFA pathways is challenging due to differences in process configurations, system boundaries, and technology readiness. VFAs produced via conventional petrochemical oxidation or fermentation routes using refined feedstocks benefit from scale, mature separations, and optimized supply chains. Current global market prices provide an effective benchmark for the cost targets that waste-derived VFA systems must approach. Presently, market prices for major VFAs span a wide range, from $1,265 t^–1^ to $7,000 t^–1^, reflecting differences in market size, purity requirements, and downstream value (Table S1). For waste-derived VFAs to be economically viable at scale, the combined costs of production, separation, and upgrading must approach these market price ranges after accounting for avoided waste treatment costs, methane abatement benefits, and potential policy incentives. While complete methanogenesis offers simpler process control and lower separation requirements, it also locks carbon into a low-value energy carrier, whereas VFA recovery enables carbon retention in higher-value chemical and material pathways that can justify additional process complexity under appropriate system conditions.

VFAs stand at the frontier of a new carbon economy that is modular, distributed, and deeply integrated with renewable energy and waste management infrastructure. As global demand for sustainable energy and chemicals accelerates, waste-derived VFAs can emerge as a flexible carbon intermediate that links waste valorization and carbon utilization across sectors. VFA recovery and upgrading systems can be scaled through modular biorefineries that colocate with municipal, agricultural, or industrial waste streams, reducing transport costs and enabling localized production of carbon-neutral intermediates. Integrating VFA into the circular economy framework can accelerate infrastructure redesign, shifting from centralized petrochemical models toward adaptive regional networks of carbon conversion hubs.

## Supplementary Material



## Notes

Calculation of projected VFA demand; calculation of global potential of recoverable VFA; and calculation of VFA allocation relative to 2050 market demand of key industrial and energy products (PDF)

## References

[1] Vázquez-Fernández A., Suárez-Ojeda M. E., Carrera J. (2022). Review about bioproduction
of Volatile Fatty Acids from wastes and wastewaters: Influence of
operating conditions and organic composition of the substrate. Journal of Environmental Chemical Engineering.

[2] Ramos-Suarez M., Zhang Y., Outram V. (2021). Current perspectives
on acidogenic
fermentation to produce volatile fatty acids from waste. Reviews in Environmental Science and Bio/Technology.

[3] Anjum A., Zuber M., Zia K. M., Noreen A., Anjum M. N., Tabasum S. (2016). Microbial production
of polyhydroxyalkanoates (PHAs)
and its copolymers: A review of recent advancements. Int. J. Biol. Macromol..

[4] Girotto F., Alibardi L., Cossu R. (2015). Food waste
generation and industrial
uses: A review. Waste management.

[5] Huq N. A., Hafenstine G. R., Huo X., Nguyen H., Tifft S. M., Conklin D. R., Stück D., Stunkel J., Yang Z., Heyne J. S. (2021). Toward
net-zero sustainable aviation fuel with
wet waste-derived volatile fatty acids. Proc.
Natl. Acad. Sci. U.S.A..

[6] Angenent L. T., Casini I., Schröder U., Harnisch F., Molitor B. (2024). Electrical-energy
storage into chemical-energy carriers by combining or integrating
electrochemistry and biology. Energy Environ.
Sci..

[7] Chen W., He P., Zhang H., Lü F. (2024). Effects of volatile fatty acids on
soil properties, microbial communities, and volatile metabolites in
wheat rhizosphere of loess. Journal of Cleaner
Production.

[8] Daramola D. A., Hatzell M. C. (2023). Energy demand of
nitrogen and phosphorus based fertilizers
and approaches to circularity. ACS Energy Letters.

[9] State and Trends of Carbon Pricing 2025; World Bank, 2025. https://hdl.handle.net/10986/43277 (accessed 2025-12-16).

[10] Arriaga M., Pinar F. J., Izarra I., Amo J. d., Vicente J., Fernández-Morales F. J., Mena J. (2025). Valorization of Agri-Food
Waste into PHA and Bioplastics: From Waste Selection to Transformation. Applied Sciences.

[11] Li L., Wang X., Miao J., Abulimiti A., Jing X., Ren N. (2022). Carbon neutrality
of wastewater treatment-A
systematic concept beyond the plant boundary. Environmental Science and Ecotechnology.

[12] Fu J., Houston L., Giordan J., Grandbois M. L., Maclachlan J., Skinner J., Cohen J., Liu J. L. (2023). Opportunities
and challenges for building market-aligned sustainable aviation fuels. ACS Energy Letters.

[13] Atasoy M., Owusu-Agyeman I., Plaza E., Cetecioglu Z. (2018). Bio-based
volatile fatty acid production and recovery from waste streams: Current
status and future challenges. Bioresource technology.

[14] Yoneda N., Kusano S., Yasui M., Pujado P., Wilcher S. (2001). Recent advances
in processes and catalysts for the production of acetic acid. Applied Catalysis A: General.

[15] Weissermel, K. ; Arpe, H.-J. Industrial Organic Chemistry, 4th ed.; Wiley-VCH, Weinheim, 2003.

[16] Playne, M. Propionic and butyric acids. Comprehensive Biotechnology; Pergamon Press, 1985; Vol. 3, pp 731–759.

[17] Lucas F. W., Grim R. G., Tacey S. A., Downes C. A., Hasse J., Roman A. M., Farberow C. A., Schaidle J. A., Holewinski A. (2021). Electrochemical
routes for the valorization of biomass-derived feedstocks: from chemistry
to application. ACS Energy Letters.

[18] Liu X., Yu X. (2020). Enhancement of butanol
production: from biocatalysis to bioelectrocatalysis. ACS Energy Letters.

[19] Kang, A. J. ; Yuan, Q. Enhanced anaerobic digestion of organic waste. Solid waste management in rural areas; IntechOpen, 2017. 10.5772/intechopen.70148.

[20] Welch A. J., Digdaya I. A., Kent R., Ghougassian P., Atwater H. A., Xiang C. (2021). Comparative Technoeconomic Analysis
of Renewable Generation of Methane Using Sunlight, Water, and Carbon
Dioxide. ACS Energy Letters.

[21] Weiland P. (2010). Biogas production:
current state and perspectives. Appl. Microbiol.
Biotechnol..

[22] Kapdi S. S., Vijay V. K., Rajesh S. K., Prasad R. (2005). Biogas scrubbing, compression
and storage. Renewable Energy.

[23] Schulte-Schulze Berndt, A. Biogas upgrading with pressure swing adsorption versus biogas reforming. Biofuels for Fuel Cells; Lens, P. , Westermann, P. , Haberbauer, M. , Moreno, A. , Eds.; IWA Publishing, 2005; pp 414–429.

[24] Appels L., Baeyens J., Degrève J., Dewil R. (2008). Principles and potential
of the anaerobic digestion of waste-activated sludge. Prog. Energy Combust. Sci..

[25] Tchobanoglous, G. ; Stensel, H. D. ; Burton, F. L. ; Wastewater Engineering: Treatment and Resource Recovery, 5th ed.; McGraw-Hill Education, New York, NY, USA, 2014.

[26] Jankowska E., Duber A., Chwialkowska J., Stodolny M., Oleskowicz-Popiel P. (2018). Conversion
of organic waste into volatile fatty acids–The influence of
process operating parameters. Chemical Engineering
Journal.

[27] Deublein, D. ; Steinhauser, A. Biogas from waste and renewable resources: an introduction; John Wiley & Sons, 2011.

[28] Wang K., Yin J., Shen D., Li N. (2014). Anaerobic digestion of food waste
for volatile fatty acids (VFAs) production with different types of
inoculum: effect of pH. Bioresource technology.

[29] Bhatia S. K., Yang Y.-H. (2017). Microbial production
of volatile fatty acids: current
status and future perspectives. Reviews in Environmental
Science and Bio/Technology.

[30] Zhou M., Yan B., Wong J. W.C., Zhang Y. (2018). Enhanced volatile
fatty acids production
from anaerobic fermentation of food waste: A mini-review focusing
on acidogenic metabolic pathways. Bioresource
Technology.

[31] Raspor P., Goranovič D. (2008). Biotechnological Applications of Acetic Acid Bacteria. Critical Reviews in Biotechnology.

[32] He G.-q., Kong Q., Chen Q.-h., Ruan H. (2005). Batch and fed-batch
production of butyric acid by Clostridium butyricum ZJUCB. Journal of Zhejiang University-SCIENCE B.

[33] Zigová J., Šturdık E., Vandák D., Schlosser (1999). Butyric acid production by Clostridium
butyricum with integrated extraction and pertraction. Process Biochemistry.

[34] Jiang L., Wang J., Liang S., Wang X., Cen P., Xu Z. (2010). Production of Butyric Acid from Glucose and Xylose
with Immobilized
Cells of Clostridium tyrobutyricum in a Fibrous-bed Bioreactor. Appl. Biochem. Biotechnol..

[35] Mitchell R. J., Kim J.-S., Jeon B.-S., Sang B.-I. (2009). Continuous hydrogen
and butyric acid fermentation by immobilized Clostridium tyrobutyricum
ATCC 25755: Effects of the glucose concentration and hydraulic retention
time. Bioresour. Technol..

[36] Canganella F., Wiegel J. (2000). Continuous cultivation
of Clostridium thermobutyricum
in a rotary fermentor system. Journal of Industrial
Microbiology and Biotechnology.

[37] Coral J., Karp S. G., Porto de Souza Vandenberghe L., Parada J. L., Pandey A., Soccol C. R. (2008). Batch fermentation
model of propionic acid production by Propionibacterium acidipropionici
in different carbon sources. Appl. Biochem.
Biotechnol..

[38] Bhatia S. K., Yang Y.-H. (2017). Microbial production of volatile fatty acids: current
status and future perspectives. Reviews in Environmental
Science and Bio/Technology.

[39] Baumann I., Westermann P. (2016). Microbial Production of Short Chain
Fatty Acids from
Lignocellulosic Biomass: Current Processes and Market. BioMed. Research International.

[40] Cheah Y.-K., Dosta J., Mata-Álvarez J. (2019). Enhancement
of Volatile Fatty Acids
Production from Food Waste by Mature Compost Addition. Molecules.

[41] Tampio E. A., Blasco L., Vainio M. M., Kahala M. M., Rasi S. E. (2019). Volatile
fatty acids (VFAs) and methane from food waste and cow slurry: Comparison
of biogas and VFA fermentation processes. GCB
Bioenergy.

[42] Kleerebezem R., van Loosdrecht M. C. (2007). Mixed culture
biotechnology for bioenergy production. Curr.
Opin. Biotechnol..

[43] Lu Y., Slater F. R., Mohd-Zaki Z. (2011). Impact of operating
history on mixed culture fermentation microbial ecology and product
mixture. Water Sci. Technol..

[44] Zhou M., Yan B., Wong J. W., Zhang Y. (2018). Enhanced volatile fatty acids production
from anaerobic fermentation of food waste: A mini-review focusing
on acidogenic metabolic pathways. Bioresource
technology.

[45] Agnihotri S., Yin D.-M., Mahboubi A., Sapmaz T., Varjani S., Qiao W., Koseoglu-Imer D. Y., Taherzadeh M. J. (2022). A Glimpse
of the World of Volatile Fatty Acids Production and Application: A
review. Bioengineered.

[46] Jomnonkhaow U., Uwineza C., Mahboubi A. (2021). Membrane bioreactor-assisted
volatile fatty acids production and in situ recovery from cow manure. Bioresour. Technol..

[47] Liu H., Han P., Liu H. (2018). Full-scale production of VFAs from sewage sludge
by anaerobic alkaline fermentation to improve biological nutrients
removal in domestic wastewater. Bioresour. Technol..

[48] Xu Q., Liu X., Fu Y. (2018). Feasibility of enhancing short-chain fatty
acids production from waste activated sludge after free ammonia pretreatment:
role and significance of rhamnolipid. Bioresour.
Technol..

[49] Yan Y., Feng L., Zhang C. (2010). Ultrasonic enhancement
of waste activated sludge hydrolysis and volatile fatty acids accumulation
at pH 10.0. Water Res..

[50] Yuan H., Chen Y., Zhang H. (2006). Improved bioproduction
of short-chain fatty acids (SCFAs) from excess sludge under alkaline
conditions. Environ. Sci. Technol..

[51] Zhao J., Wang D., Li X. (2015). Free nitrous acid serving
as a pretreatment method for alkaline fermentation to enhance short-chain
fatty acid production from waste activated sludge. Water Res..

[52] Atasoy M., Owusu-Agyeman I., Plaza E., Cetecioglu Z. (2018). Production
of volatile fatty acids from organic-rich waste streams: Current issues,
challenges, and opportunities. Bioresour. Technol..

[53] Deshmukh, G. ; Manyar, H. G. Production Pathways of Acetic Acid and Its Versatile Applications in the Food Industry. Biotechnological Applications of Biomass; IntechOpen, 2021. 10.5772/intechopen.92289.

[54] Jiang L., Fu H., Yang H. K., Xu W., Wang J., Yang S.-T. (2018). Butyric
acid: Applications and recent advances in its bioproduction. Biotechnol. Adv..

[55] Kim H. J., Choi Y. G., Kim D. Y., Kim D. H., Chung T. H. (2005). Effect
of pretreatment on acid fermentation of organic solid waste. Water Science and Technology: A Journal of the International
Association on Water Pollution Research.

[56] Pavlostathis S., Giraldo-Gomez E. (1991). Kinetics of
anaerobic treatment: a critical review. Critical
Reviews in Environmental Science and Technology.

[57] Sun S., Wang X., Cheng S., Lei Y., Sun W., Wang K., Li Z. (2024). A review of volatile
fatty acids
production from organic wastes: Intensification techniques and separation
methods. Journal of Environmental Management.

[58] Shrestha S., Pandey R., Aryal N., Lohani S. P. (2023). Recent advances
in co-digestion conjugates for anaerobic digestion of food waste. Journal of Environmental Management.

[59] Liu H., Wang J., Liu X., Fu B., Chen J., Yu H.-Q. (2012). Acidogenic fermentation of proteinaceous sewage sludge: Effect of
pH. Water Res..

[60] Neyens E., Baeyens J., Dewil R., De heyder B. (2004). Advanced sludge
treatment affects extracellular polymeric substances to improve activated
sludge dewatering. Journal of Hazardous Materials.

[61] Goud R. K., Sarkar O., Chiranjeevi P., Venkata Mohan S. (2014). Bioaugmentation
of potent acidogenic isolates: A strategy for enhancing biohydrogen
production at elevated organic load. Bioresour.
Technol..

[62] Díez M. P., Villanueva-Galindo E., Moreno-Andrade I., Díaz E., de la Rubia M. A., Mohedano A. F., Perez-Rangel M. (2025). Enhanced hydrogen
production from food waste via bioaugmentation with Clostridium and
Lactobacillus. Biomass Conversion and Biorefinery.

[63] Murali N., Srinivas K., Ahring B. K. (2021). Increasing
the Production of Volatile
Fatty Acids from Corn Stover Using Bioaugmentation of a Mixed Rumen
Culture with Homoacetogenic Bacteria. Microorganisms.

[64] De
Maayer P., Anderson D., Cary C., Cowan D. A. (2014). Some like
it cold: understanding the survival strategies of psychrophiles. EMBO reports.

[65] Pind P. F., Angelidaki I., Ahring B. K. (2003). Dynamics of the anaerobic process:
Effects of volatile fatty acids. Biotechnol.
Bioeng..

[66] Lan K., Xu S., Li J., Hu C. (2019). Recovery of Lactic Acid from Corn
Stover Hemicellulose-Derived Liquor. ACS Omega.

[67] Pervez M. N., Mahboubi A., Uwineza C., Zarra T., Belgiorno V., Naddeo V., Taherzadeh M. J. (2022). Factors
influencing pressure-driven
membrane-assisted volatile fatty acids recovery and purification-A
review. Science of The Total Environment.

[68] Asghar N., Lee H., Jang D., Jang A. (2022). Recovery of volatile fatty acids
using forward osmosis: influence of solution chemistry, temperature,
and membrane orientation. Chemosphere.

[69] Zhao H., Zhao Y., Wang Y., Xiao G., Su H. (2025). Adsorption
and High-Value Transformation of Volatile Fatty Acids from Microbial
Fermentation Products: A Review. Green Chemical
Technology.

[70] Singh R., Palar S., Kowalczewski A., Swope C., Parameswaran P., Sun N. (2023). Adsorptive recovery
of volatile fatty acids from wastewater fermentation
broth. Journal of Environmental Chemical Engineering.

[71] Tahmid M., Joo Choi H., Ganapavarapu S. T., Scott J., Hatzell M. C. (2024). Concentrating
nitrogen waste with electrodialysis for fertilizer production. Environmental Science & Technology Letters.

[72] Choi H. J., Tahmid M., Mondal S., Hatzell M. C. (2025). Concentrating Ammonia
from Wastewater with Electrodialysis. ACS Es&t
Water.

[73] Jones R. J., Massanet-Nicolau J., Guwy A., Premier G. C., Dinsdale R. M., Reilly M. (2015). Removal and recovery of inhibitory volatile fatty acids
from mixed acid fermentations by conventional electrodialysis. Bioresour. Technol..

[74] Huang C., Xu T., Zhang Y., Xue Y., Chen G. (2007). Application of electrodialysis
to the production of organic acids: State-of-the-art and recent developments. J. Membr. Sci..

[75] Redwood M. D., Orozco R. L., Majewski A. J., Macaskie L. E. (2012). Electro-extractive
fermentation for efficient biohydrogen production. Bioresour. Technol..

[76] Haflich H., Ding H., Call D. F., Coronell O. (2025). Effects of Electrodialysis
Physical and Operating Parameters on the Performance of an Anaerobic
Digestion–Electrodialysis System for Volatile Fatty Acid Production
and Recovery. ACS omega.

[77] M’Arimi M. M., Mecha C. A., Kiprop A. K., Ramkat R. (2020). Recent trends in applications
of advanced oxidation processes (AOPs) in bioenergy production: Review. Renewable and Sustainable Energy Reviews.

[78] Medeiros M. C., dos Santos E. V., Martínez-Huitle C. A., Fajardo A. S., Castro S. S. L. (2020). Obtaining high-added value products
from the technical
cashew-nut shell liquid using electrochemical oxidation with BDD anodes. Sep. Purif. Technol..

[79] Jafari M., Botte G. G. (2022). Electrochemical valorization of waste activated sludge
for short-chain fatty acids production. Frontiers
in Chemistry.

[80] Medeiros M. C., Castro S. S. L., dos
Santos E. V., Rodrigo M. A., Martínez-Huitle C. A. (2022). A proof
of concept for the electro-refinery: Selective electroproduction of
acetic acid from t-CNSL waste by using DSA electrode. Electrochem. Commun..

[81] Freitas J. M., Oliveira T. d., Munoz R. A. A., Richter E. M. (2019). Boron Doped Diamond
Electrodes in Flow-Based Systems. Frontiers
in Chemistry.

[82] Meng X., Han K. N. (1996). The principles and
applications of ammonia leaching
of metals–a review. Mineral Processing
and Extractive Metullargy Review.

[83] Zhong S., Hepworth M. T. (1995). A calculation method for determining
equilibria in
metal-ammonia-water systems. Hydrometallurgy.

[84] Hatzell M. C., Kim Y., Logan B. E. (2013). Powering
microbial electrolysis cells by capacitor
circuits charged using microbial fuel cell. J. Power Sources.

[85] Cusick R. D., Hatzell M., Zhang F., Logan B. E. (2013). Minimal RED cell
pairs markedly improve electrode kinetics and power production in
microbial reverse electrodialysis cells. Environ.
Sci. Technol..

[86] Zhang J., Wang W., You S., Qi D., Liu Z., Liu D., Ma M., Cui F., Ren N., Chen X. (2020). Photothermal
Janus Anodes: Photothermal Janus Anode with Photosynthesis-Shielding
Effect for Activating Low-Temperature Biological Wastewater Treatment
(Adv. Funct. Mater. 7/2020). Adv. Funct. Mater..

[87] Cui K., Guo K., Carvajal-Arroyo J. M., Arends J., Rabaey K. (2023). An electrolytic
bubble column with an external hollow fiber membrane gas–liquid
contactor for effective microbial electrosynthesis of acetate from
CO2. Chemical Engineering Journal.

[88] Wang H., Ren Z. J. (2013). A comprehensive
review of microbial electrochemical
systems as a platform technology. Biotechnology
advances.

[89] Aranha I. (2026). Wastewater
resource harnessing: Applications and challenges of microbial electrochemical
technologies. Microbial Electrochemical Systems
for Industrial Wastewater Treatment and Research.

[90] Hill A., Tait S., Baillie C., Virdis B., McCabe B. (2020). Microbial
electrochemical sensors for volatile fatty acid measurement in high
strength wastewaters: A review. Biosens. Bioelectron..

[91] Trivedi, P. Global Acetic Acid Market Size, Share and Growth Analysis Report, 2025–2034, Report ID PM1155; Polaris Market Research & Consulting, Inc., 2025.

[92] Propionic Acid Market Report by Application, End Use Industry, and Region 2025–2033; IMARC Group, 2024.

[93] Butyric Acid Market Report by Type (Synthetic, Bio-based), Derivative (Sodium Butyrate, Calcium Butyrate, Others), Application (Animal Feed, Chemical Intermediate, Food & Flavor, Pharmaceutical, Perfume, Others), and Region 2025–2033; IMARC Group, 2025.

[94] Valeric Acid Market - Global Industry Analysis, Size, Share, Growth, Trends, and Forecast 2024–2034; Transparency Market Research, Inc., 2024.

[95] Althaus, H.-J. ; Chudacoff, M. ; Hischier, R. ; Jungbluth, N. ; Osses, M. ; Primas, A. ; Hellweg, S. Life cycle inventories of chemicals; 2007.

[96] Budsberg E., Morales-Vera R., Crawford J. T., Bura R., Gustafson R. (2020). Production
routes to bio-acetic acid: life cycle assessment. Biotechnology for biofuels.

[97] de Jong, M. ; Bunse, M. ; Hamelinck, C. Methanol Carbon Footprint and Certification Guidance; VOF Studio Gear Up, 2022.

[98] Anastas P., Eghbali N. (2010). Green chemistry: principles
and practice. Chem. Soc. Rev..

[99] Li X., Sadiq S., Zhang W., Chen Y., Xu X., Abbas A., Chen S., Zhang R., Xue G., Sobotka D. (2021). Salinity
enhances high optically active L-lactate production
from co-fermentation of food waste and waste activated sludge: Unveiling
the response of microbial community shift and functional profiling. Bioresour. Technol..

[100] Reekie, L. ; Electricity use and management in the municipal water supply and wastewater industries; Water Research Foundation, 2013.

[101] Anaerobic Digestion Market Report—America: United States of America’s Anaerobic Digestion Industry; World Biogas Association, 2018.

[102] Gee, C. Unlocking Fertilizer’s Full Potential with Biostimulants. TIMAC AGRO USA, 2025. https://us.timacagro.com/news/plant-nutrition/unlocking-fertilizers-full-potential-with-biostimulants/ (accessed 2025-11-12).

[103] Atasoy M., Scott W. T., Regueira A., Mauricio-Iglesias M., Schaap P. J., Smidt H. (2024). Biobased short
chain
fatty acid production-exploring microbial community dynamics and metabolic
networks through kinetic and microbial modeling approaches. Biotechnology Advances.

[104] Yu X., Yin J., Shen D., Shentu J., Long Y., Chen T. (2018). Improvement
of acidogenic fermentation for volatile fatty acid production
from protein-rich substrate in food waste. Waste
Management.

[105] Li Z.-Y., Inoue D., Ike M. (2023). Mitigating ammonia-inhibition
in anaerobic digestion by bioaugmentation: A review. Journal of Water Process Engineering.

[106] Lü F., Wang Z., Zhang H., Shao L., He P. (2021). Anaerobic digestion of organic waste: Recovery of value-added and
inhibitory compounds from liquid fraction of digestate. Bioresour. Technol..

[107] Wainaina S., Lukitawesa, Kumar
Awasthi M., Taherzadeh M. J. (2019). Bioengineering
of anaerobic digestion for volatile fatty acids, hydrogen or methane
production: a critical review. Bioengineered.

[108] Botte G. G., Donneys-Victoria D., Alvarez-Pugliese C. E., Adjei J., Sahin S., Wilson N. W., Millerick K., Hardberger A., Furst A. L., Hu N. (2024). Innovative
Approach to Sustainable Fertilizer Production: Leveraging Electrically
Assisted Conversion of Sewage Sludge for Nutrient Recovery. ACS omega.

