# Supplementary material for: Waste-Based Volatile Fatty Acids for Fuel and Chemical Production
Source: ACS Energy Lett. 2026 Feb 14;11(3):2521–32. doi: 10.1021/acsenergylett.5c04340 (PMC13004434; doi:10.1021/acsenergylett.5c04340)
Supplement: Supplementary file 1 [file nz5c04340_si_001.pdf]

Supporting Information: Waste-Based Volatile Fatty Acids for Fuel and Chemical Production

Mohammed Tahmid,† Hyuck Joo Choi,† Amanda Lorraine C. Cruz,† Daniela Ferreira-Garcia,‡,¶ Ehsan Abbasi,‡,¶ Gerardine G. Botte,*,‡,¶ and Marta C. Hatzell*,§,†

†*School of Chemical and Biomolecular Engineering, Georgia Institute of Technology, 311 Ferst Drive NW, Atlanta, GA, 30332, USA*

‡*Institute for Sustainability & Circular Economy, Chemical and Electrochemical Technology and Innovation Laboratory, Texas Tech University. 2500 Broadway, Lubbock, TX, 79409, USA*

¶*Department of Chemical Engineering, Texas Tech University. 2500 Broadway, Lubbock, TX, 79409, USA*

§*George W. Woodruff School of Mechanical Engineering, Georgia Institute of Technology, 770 Ferst Drive NW, Atlanta, GA, 30332, USA*

E-mail: gerri.botte@ttu.edu; marta.hatzell@me.gatech.edu

Calculation of Projected VFA Demand

The projected VFA demand in a future year is calculated by:

$$D_{\text{future}} = D_{\text{current}} \times \left(1 + \frac{\text{CAGR (\%)}}{100}\right)^n \tag{S1}$$

where, D_{future} is the projected demand after n years, D_{current} is the current demand, and CAGR (%) is the compound annual growth rate in percent.

Table S1: CAGR and Market Price of Major Volatile Fatty Acids (2024)

Volatile Fatty Acid	CAGR (%)	Market Price (USD/t)	Reference
Acetic Acid	4.4	1,265	[1]
Propionic Acid	2.7	3,830	[2]
Butyric Acid	12.3	7,133	[3]
Valeric Acid	5.3	3,000	[4]

Calculation of Global Potential of Recoverable VFA

We calculated the global total recoverable VFA from each waste stream by:

$$R_{\mathrm{overall}} = f_{\mathrm{h}} \times Y_{\mathrm{VFA}} \times f_{\mathrm{rec}} \tag{S2}$$

where, the overall recovery, R_{overall}, is the fraction of organic carbon in a waste stream that is ultimately converted to VFAs and successfully recovered. This is determined by the product of the hydrolysis fraction, f_{h}, which represents the proportion of organic matter solubilized from the waste; the VFA yield, Y_{VFA}, which quantifies the fraction of solubilized organics converted to VFAs during fermentation; and the recovery fraction, f_{rec}, which accounts for the fraction of produced VFAs that can be captured by downstream separation processes.

Table S2: Global manure generation by major livestock categories.

Livestock Category	Manure per head (kg/day)[5]	Number of animals (billions)[6]
Cattle	35.37	1.69
Swine	8.03	0.97
Poultry	0.19	22.84

Table S3: Summary of volume of global waste streams considered for VFA recovery and representative parameters for estimating VFA recovery potential.

Waste Type	Global Volume	Unit	Organic Content Basis	System Characteristics / Feedstock Description	Conversion Parameters		Recovery Fraction (f_{rec})	Process Considerations / Rationale
					Hydrolysis Fraction (f_{h})	VFA Yield (Y_{VFA})		
**Household wastewater**	268[7]	billion m^3	TOC: 80–260 mg/L (avg. 170)[8]	Dilute, continuous global stream with high treatment coverage; major urban carbon source	0.2–0.4	0.4–0.7	0.2–0.7	Low suspended solids; readily biodegradable soluble organics dominate; hydrolysis limited by low particulate fraction.
**Industrial wastewater**	36[7]	billion m^3	TOC: 100–10,000 mg/L (avg. 2000)[9]	Produced mainly by food, pulp & paper, and dairy industries; high recoverability potential	0.3–0.7	0.4–0.8		Highly variable composition; food industry effluents yield higher conversion efficiencies.
**Municipal solid waste (MSW)**	2.01[10]	billion tons	∼50% organic fraction[10]	Organic fraction primarily food and green waste; suitable for fermentation	0.5–0.8	0.5–0.8		Rich in biodegradable food/paper waste; pretreatment (e.g., shredding, enzymatic hydrolysis) enhances conversion.
**Agricultural residues**	3.7[11]	billion tons	∼50% organic fraction[12]	Requires pretreatment; hydrolysis limited by lignin and cellulose structure	0.2–0.5	0.3–0.6		Lignocellulosic matrix restricts solubilization; thermal or enzymatic pretreatment improves yields.
**Livestock manure**	26.2	billion tons	16.8% volatile solids[13]	Abundant, stable rural carbon source with high solids and fiber content	0.3–0.6	0.3–0.7		Hydrolysis is rate-limiting; acidogenic fermentation enhanced by dilution and pH control.

Table S4: Growth rates used for projecting global VFA production and recovery potential to 2050.

Growth rate	Value (% yr^{-1})	Applied to	Justification
Population growth rate	0.63[14]	Household wastewater, municipal solid waste, agricultural residue	These waste streams scale primarily with population size, making population growth an appropriate driver.
Global potential output growth	2.7[15]	Industrial wastewater	Industrial wastewater generation increases with economic and manufacturing output, which tracks global production growth.
Livestock growth rate	1.1[16,17]	Livestock manure	Manure volumes are directly linked to livestock population; growth in livestock numbers drives future manure availability.

We calculated the future global VFA recovery potential by applying an annual percentage growth rate to the present-day value as follows:

$$R_{\text{future}} = R_{\text{current}} \times \left(1 + \frac{g_{\%}}{100}\right)^{n} \tag{S3}$$

where R_{future} is the global VFA recovery potential n years in the future, R_{current} is the current recovery potential and $g_{\%}$ is the annual growth rate expressed as a percentage (e.g., 0.63%).

Calculation of VFA Allocation Relative to 2050 Market Demand of Key Industrial and Energy Products

Table S5: Sectoral VFA allocation, conversion yields, and fraction of 2050 market demand satisfied by VFAs. Conversion yields indicate the mass of product obtained per ton of VFA (t product / t VFA).

**Market**	**VFA allocation (Mt/y)**	**Conversion yield (t product / t VFA)**	**Product mass (Mt/y)**	**2050 market demand (product, Mt/y)**	**% of 2050 demand satisfied**
Commodity Chemicals	58	1.0	58	58 (VFA commodity chemicals)	100%
Fuels	37	0.7	26	26 (SAF[18])	100%
Polymers	266	0.8	213	309 (bio & recycled plastics[19,20])	69%
Agriculture	20	1.0	20	20 (fertilizer additives[21])	100%
Energy Storage	200	0.6	120	355 (clean H_2 for energy storage[22])	34%
**Total**	581	—	437	—	—

References

(1) Polaris MarketResearch & Consulting, I. *Global Acetic Acid Market Size, Share & Growth Analysis Report, 2025-2034*; 2025; Report ID: PM1155; Base Year: 2024; Historical Data: 2020-2023; Forecast Period: 2025-2034.

(2) Group, I. *Propionic Acid Market Report by Application, End Use Industry, and Region 2024-2032*; 2024; Global propionic acid market size reached US 1.8*billionin*2023, *forecasttoreachUS*2.3 billion in 2032 at a CAGR of 2.7%.

(3) Group, I. *Butyric Acid Market Report by Type (Synthetic, Bio-based), Derivative (Sodium Butyrate, Calcium Butyrate, Others), Application (Animal Feed, Chemical Intermediate, Food Flavor, Pharmaceutical, Perfume, Others), and Region 2025-2033*; 2025; Global butyric acid market size reached USD 263.5 Million in 2024, projected to reach USD 749.0 Million by 2033 at a CAGR of 11.69%.

(4) Research, T. M. *Valeric Acid Market – Global Industry Analysis, Size, Share, Growth, Trends, and Forecast 2024-2034*; 2024; Market valued at US$ 186.3 Mn in 2023 and projected to reach US$ 396.1 Mn by 2034 at a CAGR of 7.2%.

(5) Díaz-Vázquez, D.; Alvarado-Cummings, S. C.; Meza-Rodríguez, D.; Senés-Guerrero, C.; de Anda, J.; Gradilla-Hernández, M. S. Evaluation of biogas potential from livestock manures and multicriteria site selection for centralized anaerobic digester systems: The case of Jalisco, Mexico. *Sustainability* **2020**, *12*, 3527.

(6) Association, W. B. *Anaerobic Digestion Market Report — America: United States of America's Anaerobic Digestion Industry*; Market Report, 2018; Accessed: 2025-11-12.

(7) UN-Habitat, W. Progress on the proportion of domestic and industrial wastewater flows safely treated–Mid-term status of SDG Indicator 6.3. 1 and acceleration needs, with a special focus on climate change, wastewater reuse and health., in. *United Nations Human Settlements Programme (UN-Habitat) and World Health Organization (WHO)* **2024**,

(8) Kiepper, B. H. *Understanding Laboratory Wastewater Tests: I. Organics (BOD, COD, TOC, O&G)*; 2010.

(9) Berger, B. B. Control of organic substances in water and waste water. **1987**,

(10) Chen, Y.; Zhang, X.; Chen, Y. Propionic acid-rich fermentation (PARF) production from organic wastes: A review. *Bioresource Technology* **2021**, *339*, 125569.

(11) Scott Bentsen, N.; Nilsson, D.; Larsen, S.; Stupak, I. Agricultural residue for Energy in Sweden and Denmark—Differences and Commonalities. *IEA Bioenergy* **2016**, *43*, 1–28.

(12) Uwamahoro, H.; Kpomblekou-A, K.; Mortley, D.; Quarcoo, F. Organic vegetable crop residue decomposition in soils. *Heliyon* **2023**, *9*.

(13) Onwosi, C. O.; Ozoegwu, C. G.; Nwagu, T. N.; Nwobodo, T. N.; Eke, I. E.; Igbokwe, V. C.; Ugwuoji, E. T.; Ugwuodo, C. J. Cattle manure as a sustainable bioenergy source: Prospects and environmental impacts of its utilization as a major feedstock in Nigeria. *Bioresource Technology Reports* **2022**, *19*, 101151.

(14) Suzuki, E.; Pirlea, A. F. World Population Day: trends and demographic changes. 2025; `https://blogs.worldbank.org/en/opendata/world-population-day--trends-and-demographic-changes`, Accessed: 2025-11-13.

(15) OECD *OECD Global Long-Run Economic Scenarios: 2025 Update*; 2025; Accessed: 2025-11-13.

(16) Thornton, P. K. Livestock production: recent trends, future prospects. *Philosophical Transactions of the Royal Society B: Biological Sciences* **2010**, *365*, 2853–2867.

(17) Dohlman, E.; Hansen, J.; Chambers, W.; Committee, I. A. P.; others USDA Agricultural Projections to 2034. **2025**,

(18) Wang, B.; Ting, Z. J.; Zhao, M. Sustainable aviation fuels: Key opportunities and challenges in lowering carbon emissions for aviation industry. *Carbon Capture Science & Technology* **2024**, *13*, 100263.

(19) Dokl, M.; Copot, A.; Krajnc, D.; Van Fan, Y.; Vujanović, A.; Aviso, K. B.; Tan, R. R.; Kravanja, Z.; Čuček, L. Global projections of plastic use, end-of-life fate and potential changes in consumption, reduction, recycling and replacement with bioplastics to 2050. *Sustainable Production and Consumption* **2024**, *51*, 498–518.

(20) Recycling, E. Recycling and bioplastics: Where we are, and what we're up against. https://envirobank.com.au/blog-demand-for-plastic-and-the-route-to-net-zero-plastics/, 2022; Accessed: 2025-11-13.

(21) Fact.MR Fertilizer Additives Market: Analysis by Anti-caking Agents, Dust Suppressors, Drying Agents, and Granulating Agents from 2023 to 2033. https://www.factmr.com/report/3052/fertilizer-additives-market, 2023; Accessed: 2025-11-13.

(22) Sabgir, C. Power-to-X's Role in the Hydrogen Economy. https://fchea.org/power-to-xs-role-in-the-hydrogen-economy/, 2025; Accessed: 2025-11-13.
